# Psychophysiological Response According to the Greenness Index of Subway Station Space

**DOI:** 10.3390/s21134360

**Published:** 2021-06-25

**Authors:** Won-Ji Kim, Tae-Kyung Lee

**Affiliations:** Department of Interior and Environmental Design, Pusan National University, Busan 46241, Korea; flowersk@pusan.ac.kr

**Keywords:** interior landscape, greenness index, subway stations space, electroencephalogram

## Abstract

This study proposed a plan for implementing a pleasant and healthy indoor landscape in subway station space. To this end, it established a 3D landscape model of the subway interior by reviewing previous studies on indoor landscape and the greenness index of indoor spaces. Moreover, it investigated and analyzed psychophysiological responses of users to environmental indoor landscape design in subway station space. Subway stations were classified as underground subway stations and ground subway stations according to the presence of natural light inflow. The greenness index of indoor spaces was also divided into four types of 0%, 10%, 15%, and 20%. Through this process, eight 3D landscape models of the subway interior were implemented. In addition, this study investigated psychophysiological responses of 60 male and female adults in their 20 s and 30 s using the models implemented. The investigation result was analyzed based on a frequency analysis, the χ^2^ test, *T*-test, one-way analysis of variance, and multidimensional scaling, which were performed in SPSS Statistics 25. The results of this study can be summarized as follows. First, physiological responses of research subjects were analyzed based on their prefrontal α wave asymmetric values. The analytic result showed that the environment where interior landscape was adopted produced more positive effects than the environment where interior landscape was not adopted. Second, psychological responses of research subjects were examined based on their greenness index preference, awareness of interior landscape area, attention restoration effect, and space images. The analytic result indicated that, among eight 3D landscape models of the subway interior, they preferred the model with the greenness index of 15% for underground subway stations. In addition, they preferred the model with the greenness index of 10% the most for ground subway stations.

## 1. Introduction

### 1.1. Research Background and Purpose

Subways are being used for public transportation to address space shortage attributed to rapid economic growth and urbanization. In South Korea, subway line 1 was first opened in Seoul in 1974, and Busan subway line 1 was opened in 1985. Subsequently, subway line 4 (Busan-Gimhae Light Rail Transit) and Donghae line were opened in Busan. The construction of a metropolitan rapid transit network is currently in progress, with the aim of building seven additional lines in the future. In addition, the number of Busan metropolitan railway daily transit passengers has increased from 98,000 in 1985 to 753,000 in 2010 and 938,000 in 2019.

As subway spaces are expanded and the number of subway users increase, the environmental designs of subway station spaces have garnered significant research attention. Subway station users travelling via new sections and existing lines have negatively responded to the existing subway station, which is relatively old and unclean, and have advocated the creation of a cleaner and healthier space [1]. Furthermore, the Ministry of Environment has prepared guidelines for developing ecological landscape of underground spaces according to Article 43 (2) of the Natural Environmental Conservation Act in 2009 and prescribed requirements to effectively create a green belt in underground spaces such as subways, which are regarded as important living spaces in the city [2]. This shows that there is a need to improve existing closed and complex subway stations and propose strategies to modify subway stations for creating pleasant environments.

Accordingly, the biophilic approach—the concept of bringing back nature in the city—has emerged as a natural and holistic approach in urban environments through design and governance in recent years [3]. It is the most well-known greening and design approach among the public [4]. The term “biophilic approach” was invented by E.O. Wilson, who was working as a professor at Harvard University, in 1984. Existing studies on the biophilic approach reported that exposing people to natural environments had positive effects on reducing their stress level, recovering their mental fatigue, and increasing their physical functions and abilities [5,6,7,8]. In this regard, green spaces ensured in indoor environments can be advantageous for enhancing the physical and psychological health of people.

The introduction of plants to underground spaces has been passively conducted so far, despite its advantages in enhancing aesthetics while serving decorative, constructive, and psychological and emotional functions. However, the Ministry of Environment has recently encouraged the use of green spaces in underground areas by laying out the general guidelines for designing urban railway platforms of subway stations with regard to four categories: functionality, convenience, environmental friendliness, and aesthetics. In the United States, the WELL Building Standard was developed in 2012, which emphasizes accessibility to nature for the health of people in buildings. Modern people who spend a considerable amount of time inside everyday [9] are interested in creating a pleasant and healthy environment, and their desire to introduce natural elements into interior spaces, as primitive elements for psychological stability and relaxation, is increasing [10,11,12,13]. Furthermore, subway users exhibit various use patterns apart from transportation, including meeting and resting; therefore, they are recognizing the necessity of environmental improvement through greening.

Because subway station spaces applying interior landscape design resembled interior landscape spaces, a decrease in the concentration of dust and formaldehyde and an increase in humidity by approximately 3 to 4% were observed in these spaces [14]. It was also found that an increase in the greening area on indoor walls had significant effects on reducing indoor temperature, PM1, and PM10 [15]. Moreover, psychological restoration effects in subway station spaces applying interior landscape design were greater—by twice or more—than those in subway stations spaces not applying interior landscape design, on average [16].

Thus, by developing a pleasant interior landscape in subway stations that play a key role in city transport operations, various positive effects are expected including the creation of spaces and the improvement in interior environment quality, energy saving, and emotional and psychological stability of users. 

The Gare de Lyon in Paris and the Lowline Project in New York City are case studies of interior landscaping in underground areas in which various interior landscape designs have been implemented, such as the “remote skylights” system delivering natural sunlight to trees and grass underneath city streets and a water supply system using fog as a water source. In South Korea, transportation authorities are attempting to improve the interior spaces of subway stations by applying new environmental designs, such as the installation of an LED vegetable garden, wall displays, and the construction of ceramic wall paintings [17]. Recently, various efforts are being made to design an interior landscape in some subway stations at Seoul using new technologies, including a remote skylight system to control natural sunlight that has been employed by the Lowline Project in New York, smart farms, and plant-based biofilters. As such, new designs and technologies are being introduced to create a pleasant subway station spaces in Seoul as well as the world-centered cities. 

Physical environments in subway station spaces vary according to the characteristics of these spaces, such as the presence of natural light inflow, the area of a subway station, floor height, and degree of congestion [18]. For this reason, subway station spaces should be classified according to their physical environments. Accordingly, an appropriate interior landscape plan considering these types of subway station spaces is required.

Accordingly, the following research questions are presented: How can interior landscapes be designed to enhance the environmental landscape of subway stations as well as user satisfaction? How much interior space is required to achieve effective spatial, economic, and aesthetic satisfaction? How will subway users react to the interior landscape of subway stations? 

People can perceive stimuli coming from interior landscapes, such as a visual stimulus (e.g., a green view), an olfactory stimulus (e.g., the scent of flowers), and an auditory stimulus (e.g., the sound of leaves shaking), based on their five senses. Among these senses, the visual sense is regarded as the most crucial sense; visual perception, related to 2D or 3D shapes, colors, and texture, accounts for 80% of environmental perception [19,20].

The greenness index (GI) reflects the amount of green space observed by an individual as compared to the entire landscape; this index reflects the amount, level of recognition, and satisfaction associated with green space [21]. The GI of interior spaces has a significant effect on the visual preference and satisfaction of space users. According to previous studies on the GI, it is closely related to psychological stability and pleasantness [16,22,23,24,25,26,27]. However, existing studies have established a range of green belts as a part of the city landscape, while focusing on the psychological impact, exterior GI preferences, and promotions strategies associated with the GI. Conversely, the preferred GI and interior landscape design were proposed, based on the response to the GI; subsequently, commercial spaces and workspaces were targeted [16,28,29]. Therefore, to improve the environment of subway stations, users’ responses to the interior GI and preferences are required as reference data. Furthermore, by establishing interior landscape models of subway stations, creating 3D videos, and objectively measuring the response to the GI, determining the GI for 3D environments in a 2D aspect was attempted.

Therefore, a 3D landscape model of the subway interior was established in this study. Further, the users’ psychological and physiological responses to the interior landscape environment and GI of a subway station were analyzed with the objective of proposing pleasant and healthy interior landscape strategies for subway stations.

The findings of this study are expected to assist in planning and improving the interior landscape of subway stations globally. Furthermore, the findings of this study can be useful in practically analyzing the amount and recognition level of green belts. This is accomplished by providing human perspectives of interior spaces and utilizing the evaluation method of the GI via a 3D model, unlike the existing 2D measurements.

### 1.2. Research Scope and Methods

**Figure 1:**
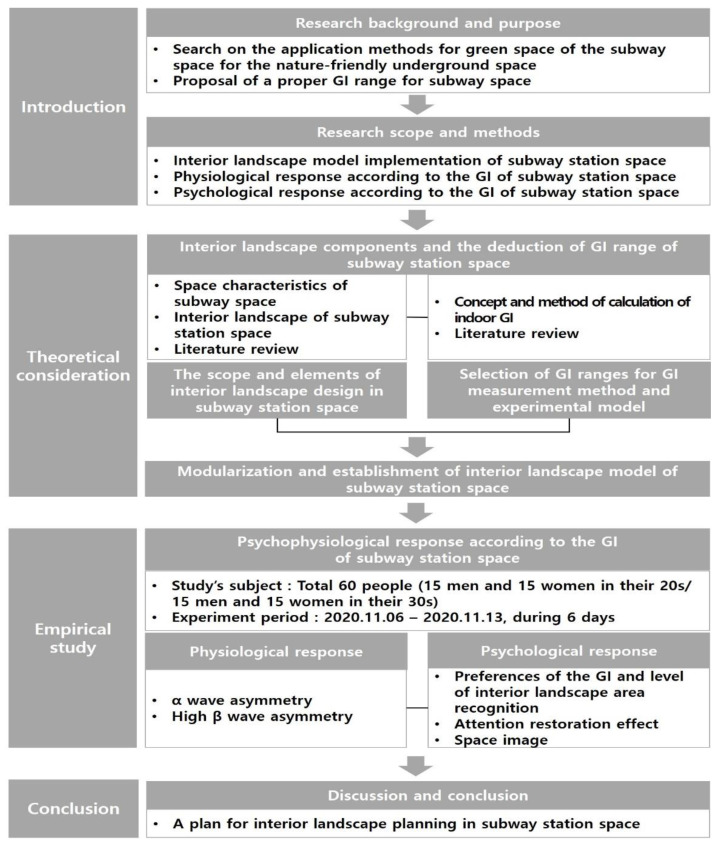
Research Flow of Study.

This study proposed a plan for implementing a desirable interior landscape in subway station spaces to enhance environmental conditions and increase user satisfaction. To this end, we developed 3D landscape models of the subway interior and investigated the psychophysiological responses of research subjects based on the models (Figure 1).

Specifically, we reviewed previous studies and classified subway stations as ground subway stations where natural light can be introduced and underground subway stations where natural light cannot be introduced based on the review result. Moreover, existing methods for applying interior landscape in subway station spaces were analyzed based on garden style, planter style, and wall style. Thus, arrangement was performed based on a combination of garden and wall styles, which did not disturb the traffic of subway users. The GI index was classified as 0%, 10%, 15%, and 20%. Through this process, eight 3D landscape models of the subway interior were implemented.

Furthermore, we analyzed psychophysiological responses of 60 male and female adults in their 20 s and 30 s, who were the main subway users, based on the 3D landscape models implemented. To analyze physiological responses of subjects, it conducted an experiment on measuring their brainwaves according to the level of the GI and 3D landscape models. To analyze psychological responses of subjects, it carried out a survey on their preference for a 3D landscape model, their perception of interior landscape area, their evaluation of subway station space images, and attention restoration effect.

## 2. Materials and Methods

### 2.1. Modularization and Establishment of Interior Landscape Model of Subway Station Space

Based on the results of a previous study that investigated the interior landscape status of subway stations [21], it was identified that spaces where the interior landscape is installed are all waiting rooms regardless of space type. Furthermore, based on the physical environment of interior landscape space, it was found that there is a significant difference in the area and floor height of the waiting rooms. In terms of the floor height of the waiting rooms, there is a difference between the ground and underground subway station, and the floor height for the ground subway station is designed to be high to stimulate the inflow of natural light. Therefore, the representative space of the subway interior landscape model was selected to be the waiting room. The interior landscape models were established after separating the ground subway station, where natural lighting is enabled, and underground subway station, where natural lighting is not introduced, to evaluate the GI of different physical environments (Table 1). 

To test and analyze the psychophysiological responses of research subjects with the presence of natural light inflow and a different GI, the area and floor height of the waiting rooms were maintained at 4958.94 m^2^ and 3 m, respectively. The sunlight was set at 6500 K, which is the peak daylight hour; illumination intensity of subway station space was 500 lx [30]; and the walls, floor, and ceiling were given an achromatic color to easily recognize the presence of an interior landscape in the space. 

To simulate a subway waiting room for evaluating the GI, Auto Cad 2020, SketchUp 2019, and Twin motion 2020 programs were used, and a waiting room area of 4958.94 m^2^ (room height of 3 m and volume of 14,876.81 m^3^) was used, considering the area mean and median values of the Busan subway waiting room.

To simulate the user walking from the subway station entrance to the square, passage, and ticket gate, a 30 s (30 s) video (considering the brainwave testing time) was used. Initially, the entire waiting room was the interior landscape space; however, because the video used in the experiment was limited to 30 s, the interior landscape of the waiting room space used was 1982.11 m^2^ (5946.33 m^3^) (excluding staircases and workspace). This granted users a realistic experience. During the experiments, subjects were informed that the interior landscape was applied to the entire subway waiting room, including four models that altered the interior landscape. 

The video was within 20° from the horizontal line at a point 1.5 m above ground based on the GI measurement to ensure subjects have a clear view of the interior landscape. The resolution of the video was 1920 × 1080 (2K Full HD). 

To view the GI, four representative spaces, namely, the entrance, square, passage, and ticket gate on the way from the waiting room to the platform, were selected as sample positions. The angle was adjusted to observe the interior landscape arrangement (to avoid obstruction by pillars), based on a previous study [16] that investigated the interior GI.

For the interior landscape design, the garden style, identified as the representative interior landscaping technique for the subway station in a previous study [21], was selected as the standard interior landscape model. The experiments were conducted in a mixed style, placing the pillars and walls at the center (planter style) and modularizing the interior landscape design to minimize pedestrian interference when using the subway station space. A previous study [31] claimed that a two-column plantation is more effective than a one-column plantation when trees are planted in the garden-style; therefore, the two-column plantation was utilized. According to the results of a previous study [16], the landscape formed of a flat plane is preferred in the planter style (pillar/wall); the plantation was either used or excluded depending on the GI using a 2 m wall width and one pillar as standard.

Sculpture and topography libraries in the Twinmotion program were used for the plants used in the interior landscape models. Five different types of plants with soft leaf shapes [22] were selected according to findings from previous studies among green foliage plants widely utilized in case studies, which are identified to improve the psychological and physiological health of humans compared with plants of other colors [16,31]. 

**Figure 2:**
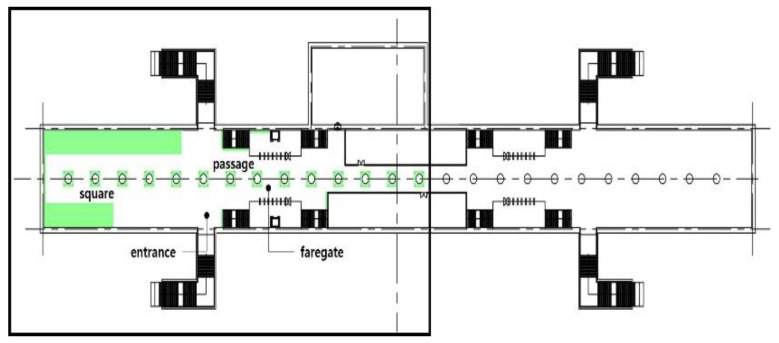
The Plan and Interior Landscape Composition Range of Subway Station Interior Landscape Model.

The volume of plants applied to the interior landscape models of underground subway station space was 236.66 m^3^ (3.98% of the subway station area (volume)) for a GI of 10%, 648 m^3^ (10.90%) for a GI of 15%, and 721.45 m^3^ (12.13%) for a GI of 20%: for ground subway stations, 235.13 m^3^ (3.95%) for a GI of 10%, 646.22 m^3^ (10.87%) for a GI of 15%, and 721.40 m^3^ (12.13%) for a GI of 20% (Figure 2). When organizing the interior landscape models for underground/ground subway station space, physical environment differences were observed for the windows and passages towards the platform (stair wall), and a permissible difference in the standard GI by each interior landscape design was accommodated.

### 2.2. Composition of Research Tools

#### 2.2.1. Brainwave Measurement Tool

An electroencephalogram (EEG) is a test for clinically detecting brain diseases, which has been used to diagnose epilepsy, sleep disturbance, comas, and encephalopathy in medical fields and perform research on neuroscience, cognition, physiology, and psychology in academic fields [32]. Accordingly, various studies [33] have been conducted to quantify inner responses to external stimulus as biosignals, including those on verifying effects of forest and gardens on reducing stress [34,35], identifying a difference in color emotions [31,36], and examining physiological effects of the fragrance of plants [35].

In terms of the frequency range of brainwaves, α wave is in the range of 8–13 Hz and denotes a comfortable state such as an emotional break or rest [37,38]. A high β wave (H.β) is in the range of 20–30 Hz and denotes awareness due to stress, such as tension and anxiety, and it occurs continuously [34,39,40].

The prefrontal cortex reflects asymmetric characteristics because of the functional differentiation of the left brain and right brain [41]. In this study, an analysis was conducted, emphasizing the absolute power values of α wave and high β wave to identify the asymmetric characteristics of the prefrontal cortex for the interior landscape model with different GI. The left and right asymmetric analyses of brainwaves were conducted by calculating the difference obtained after subtracting the left prefrontal (Fp1) frequency value from the right prefrontal (Fp2) frequency value of the absolute power value. As the α wave is inversely proportional to the level of brain activation [28], if the prefrontal α wave asymmetric value is positive (+), it implies that the left prefrontal cortex has been activated, which is related to a positive emotional state. However, if the prefrontal α wave asymmetric value is negative (−), it means the right prefrontal cortex has been activated, which is related to a negative emotional state [42,43,44,45].

The effect of interior landscape installation and GI on prefrontal brainwave activities while watching the interior landscape model video for 8 min was investigated. The Neuro Harmony system, manufactured by Panaxto sand NeuroSpec 2.4 programs, was used to measure the EEG of the prefrontal cortex. The Neuro Harmony System showed a correlation of 0.916 (*p* < 0.001) for left and right α, β, and θ waves with Grass System (USA), a well-known brainwave measuring instrument; thus, its reliability has been verified [39].

The corresponding equipment was a two-channel brainwave measuring instrument and three dry electrodes plated with pure gold to measure the real-time brainwave activity of the left and right brains. This method has simple measurement due to its hairband form, and it is agreeable to the research participants. Fp1 (left) and Fp2 (right) of the prefrontal cortex were set as active electrodes, Fpz (center) was set as the ground electrode, and the earlobes were set as reference electrodes. In terms of the frequency range of brainwaves, α wave is in the range of 8–13 Hz and measures a comfortable state such as an emotional break or rest [37,38], and high β wave (H.β) is in the range of 20–30 Hz and measures awareness due to stress, such as tension and anxiety, and it continuously occurs [34,39,40].

The occipital lobe is the area that primarily acquires visual information from the cerebrum, and the prefrontal lobe is the cortex, which analyzes color information obtained and is involved in a decision-making process. For this reason, this study used EEG values of the prefrontal lobe for analysis [42,43].

#### 2.2.2. Questionnaire Development

The preference for interior landscape models according to the GI of subway stations was investigated by preparing a questionnaire on the interior landscape model preference, attention restoration effect, and space image. In that process, the questions were reconstructed based on previous studies on the GI and interior landscaping, as well as guidelines for designing urban railway stations, amenities, and transfer facilities [16,21,22,23,29,46,47,48,49,50].

The questionnaire comprised 118 questions (GI of 0%: 14 questions each and GI of 10%, 15%, and 20%: 15 questions each) on the preference for interior landscape models. For the interior landscape model preference eight questions (one question each), level of recognition of the interior landscape area six questions (GI of 10%, 15% and 20%: one question each), attention restoration effect 56 questions (seven questions each), and space image questions 48 questions (six questions each), and a 5-point Likert scale was employed.

Subjects were asked to form questionnaires after watching videos on 3D landscape models of the subway interior according to the GI level. To analyze their preference for 3D landscape models of the subway interior, they watched videos on each 3D landscape model and expressed their preference for the corresponding interior environment. In terms of their perception of interior landscape area, they provided responses on the area of interior landscape installation that they perceived for each 3D landscape model. With regard to space images, the SD measurement method was utilized to analyze their perception of space images of each 3D landscape model.

The answers for interior landscape model preferences were: “not preferred at all (1 point)”, “not preferred (2 points)”, “moderate (3 points)”, “preferred (4 points)”, and “strongly preferred (5 points).” The answers for the level of recognition of the interior landscape area were: “too low (1 point)”, “low (2 points)”, “suitable (3 points)”, “high (4 points)”, and “very high (5 points).” The answers for interior landscape model recognition level of the interior landscape area were: “strongly disagree (1 point)”, “disagree (2 points)”, “normal (3 points)”, “agree (4 points)”, “strongly agree (5 points)”.

The attention restoration effect is subjectively measured based on the level of attention capacity, which is estimated to be restored when a person belongs to or looks at a certain environment [16]. Previous studies measured the attention restoration effect by showing slides on the natural or urban landscape to research subjects and asking them about the possibility of attention restoration [16,46,51]. The attention restoration effect can be measured based on recovery scales. The recovery scales consisted of seven items (coming to rest, renew energy, become myself again, lose all tension, order my thoughts again, put everything behind me, regain the ability to concentrate) [46].

The original recovery scales were based on a 7-point scale ranging from “not at all (1 point)” to “the very much, for the affective states; very well, for the behavioral items (7 points).” However, in this study, a 5-point Likert scale was utilized to facilitate a more convenient comparison of preferences of research subjects for 3D landscape models of the subway interior, their perception of the area, and their space image evaluation.

The answers for the attention restoration effect were, “not at all (1 point)”, “no (2 points)”, “moderate (3 points)”, “yes (4 points)”, and “absolutely (5 points).” The attention restoration points were calculated by summing up the points of seven attention restoration questions and calculating the average. 

Regarding questions on the space images, the SD (semantic differential) scale was used for the analysis. Contents of design guidelines for urban railway stations transfer facilities convenience facilities and previous studies [21,22,23,29,47,48] were referred to. Six pairs of words describing the image, namely “dark–bright”, “uncomfortable–comfortable”, “not beautiful–beautiful”, “unpleasant–pleasant”, “unharmonious–harmonious”, “un-environmentally friendly–environmentally friendly” were employed. According to the SD measurement method, opposite adjectives were marked at both ends: “strongly”, scored “1 point and 5 points, “disagree” scored 2 points and 4 points, and “normal” scored 3 points.

Before the correspondence analysis, preference, level of interior landscape area recognition, attention restoration effect, and space image measured as continuous variables (interval ratio scale) were recoded to discrete variables (nominal scale). As such, “not preferred at all (1 point)” and “not preferred (2 points)” were recoded to “not preferred GI (1)”, “moderate (3 points)” was recoded to “moderate GI (2)” and “preferred (4 points)” and “strongly preferred (5 points)” were recoded to “preferred GI (3).” For the level of interior landscape area recognition, “too low (1 point)” and “low (2 points)” were recoded to “small landscape area (1)”, “suitable (3 points)” was recoded to “suitable landscape area (2)”, and “high (4 points)” and “very high (5 points)” were recoded to “large landscape area (3).” For attention restoration effect, based on the total score (35 points), “7–20 points” was recoded to “low attention restoration effect (1)”, “21–27 points” was recoded to “moderate attention restoration effect (2)”, and “28–35 points” was recoded to “high attention restoration effect (3).” For space image, “1–2 points” were recoded to negative space image (e.g., “dark” for “bright–dark”), “moderate (3 points)” was recoded to “moderate (2)”, and “4–5 points” was recoded to positive space image (e.g., “bright” for “bright–dark”). To identify the relevance of interior landscape models providing a positive effect to research subjects among these, only the items with positive content among psychological response (“preferred GI (3)”, “suitable landscape area (2)”, “high attention restoration effect (3)”, “bright (3)”, “comfortable (3)”, “beautiful (3)”, “pleasant (3)”, “harmonious (3)”, and “environmentally friendly (3)”) were selected for analysis.

### 2.3. Experimental Method

The experiments were conducted after approval by the bioethics committee of Pusan National University in South Korea. According to the 2015 South Korea census, people in their 20 s (30%) and 30 s (25%) mainly used the subway and transit systems for work and school commutes. Thus, these age groups were selected as the study subjects, with 60 people participating in the study (30 men and 30 women) (Table 2). The experiments were conducted for approximately six days—from 6 November to 13 November 2020.

In terms of statistical calculation, the sample mean distribution approximates normal distribution as the sample size of population increases, following central limit theorem; thus, if the sample size exceeds 30, it approaches a normal distribution pattern regardless of the population distribution. Therefore, in this study, the number of samples of one group was set to 30 to ensure reliability. 

Prior to the EEG experiment, research subjects were informed of this research, research participation methods, and the formation of the agreement. Approximately 10 min were required to complete these procedures.

The brainwave measurement was conducted on the same group as the psychological response investigation. Participants’ brainwaves were measured eight times while observing the interior landscape models: once with closed eyes and seven times during the break time. Furthermore, each participant’s brainwaves were measured for 480 s, with 30 s allocated for a single measurement. Then, a survey on the eight 3D landscape models implemented was conducted for approximately 10–15 min. In total, 18–23 min was required, including the amount of time required for conducting the brainwave measurement experiment and survey. The study subjects were selected based on those who consented to the experiments and did not have brain diseases or mental illness, ophthalmologic diseases or a visual impairment, heart-related diseases, did not take medicine for underlying conditions such as diabetes, and had normal blood pressure. 

Prior to experiments, the research objective and methodology, experimental contents, and required time were explained to the selected research subjects. Alcohol consumption was prohibited for two days before the experiment, and sufficient sleep was required the day before the experiment. Caffeinated drinks and smoking, which could affect the autonomic nervous system and sensitivity, were prohibited for 2.5 h before the experiment to control the effect of these actions on experimental results [21,36,52]. 

On the day of the experiment, color blindness and color weakness were identified using the “Korean color test table”, and the experimental procedure was explained along with instructions on opening and closing eyes. Moreover, mobile phones and jewelry were restricted to prevent the interruption of electromagnetic waves. Brainwaves were measured after excluding conditions that could cause artifact by awakening the subjects’ attention, such as yawning or blinking, to minimize the movement in a comfortable position [39]. 

**Figure 3:**
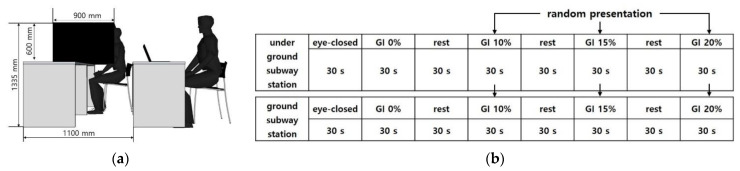
EEG Experimental Environment Configuration and Experimental Method: (**a**) EEG Experimental Environment Configuration; (**b**) EEG Experimental Environment Experiment Sequence Diagram.

An experimental booth with a width of 900 mm, length of 900 mm, and height of 1335 mm was installed (Figure 3). The monitor was fixed at eye level, 1 m from the participant. A 27-inch monitor was used, and the resolution was set at 1920 × 1080.

With regards to the psychological effects on test subjects, the external light was blocked using a blackout curtain, and fluorescent lights installed on the ceiling were turned on [53]. The effect of noise from the chair where the test subject sat was minimized by setting the criterion as below 40 dB, and an interior temperature of 22 ± 3 °C and humidity of 50 ± 5% RH [40] was fixed. The same laboratory facilities and environmental conditions were provided to all the participants. 

The EEG for interior landscape models with different GI was measured by establishing a stable state baseline with participants closing their eyes for 30 s. Then, the EEG was measured when stimulating interior landscape models with different GI for 30 s each. A break (30 s for each) was given after watching each of the eight interior landscape models to prevent a lack of concentration and tedium. The video with a GI of 0% was first presented to compare the effect of the presence of an interior landscape, and then the models with a GI of 10%, 15%, and 20% were randomly presented (Figure 3). Approximately 10–15 min was required to complete the survey.

The brainwave signal was first converted to the power value through fast Fourier transform (FFT), which interprets complex waves into simple waves before converting it to a text file (txt). One-way analysis of variance (ANOVA) was performed using SPSS Statistics 25, and the significant values of each GI were deduced based on significance probability.

Frequency analysis, χ^2^ test and *T*-test, one-way ANOVA, and MDS (Multidimensional Scaling) correspondence analysis utilizing SPSS Statistics 25 were employed on the data. When the one-way ANOVA is performed, a post-hoc test analysis is required according to the test result for homogeneity of variances. In this study, Duncan was used to perform a post-hoc test analysis when the assumption of homogeneity of variance was satisfied based on the *p*-value of the F value being 0.05 or higher in the test result for homogeneity of variances. On the other hand, the Games–Howell test was used to perform a post-hoc test analysis when the assumption of homogeneity of variance was not satisfied based on the *p*-value of the F value being 0.05 or below in the test result for homogeneity of variances. When the homogeneity of variance is assumed, Tukey’s HSD test, the Schaffe method, and the Duncan multiple range test are more frequently preferred for the multiple comparison procedures [54]. Among these methods, the Duncan multiple range test compares the ends of the mean of k group(s) adjacent to each other in stages. It shows a stronger ability for separating groups than Tukey’s HSD test and the Schaffe method. In addition, it controls the error rate related to test sets, although it performs comparison based on phased comparison order such as the Student–Newman–Keul method [55]. The purpose of the ANOVA in-depth analysis is to see if there is a statistically significant difference between groups and which group on a given issue is significant. Therefore, a Duncan post-hoc test is used to evaluate the actual cluster level [56].

## 3. Results

### 3.1. Physiological Response to Different GI for the Interior Landscape of Subway Station Space

#### 3.1.1. α Wave Asymmetry with Different GI for the Interior Landscape of Subway Station Space

One-way ANOVA was conducted to investigate the α wave asymmetry of interior landscape models with a different GI of subway station space (Table 3) and closed eyes was showed 0.45 (SD = 0.98). There was a difference in the α wave asymmetry value depending on the presence of installed interior landscape, suggesting that the installation of interior landscapes could facilitate a psychologically stable state compared to space without an interior landscape. The test results for homogeneity of variances showed that the *p*-value of the F value was 0.05 or higher (*p*-value of underground subway station = 0.65, *p*-value of ground subway station = 0.83) and that the assumption of homogeneity of variance was satisfied. For this reason, Duncan was utilized to conduct a post-hoc test analysis.

Meanwhile, the underground space with a GI of 0% had the lowest α wave asymmetry value of 0.47 (SD = 0.90), while the ground GI of 0% had an α wave asymmetry value of 0.64 (SD = 0.8). These differing values indicate that for an environment with no interior landscape model installed, the α wave asymmetry value on the ground where natural light could be introduced was higher than that of the underground. These results are similar to previous studies [13], which claim that among subjects exposed to roses, the high-frequency component of heart rate variability was significantly higher than in controls. The results of brainwave change with different leaf shapes, sizes, and ear-types of foliage plants in this study were in line with the results of a previous study [22]. An existing study [21] reported no difference in physiological responses according to the GI (5%, 20%, 50%, and 80%) of indoor spaces. Similar to the aforementioned study, this study found no significant difference in models according to the GI of 10%, 15%, and 20%. Therefore, it was concluded that the presence of plants rather than the quantitative increase in plants could positively affect mental relaxation and psychological stability.

#### 3.1.2. High β Wave Asymmetry with Different GI for the Interior Landscape of Subway Station Space

One-way ANOVA conducted to investigate the high β wave asymmetry with a different GI of subway station space (Table 4) was 0.07 (SD = 0.24) with closed eyes; the underground GI of 0%, 10%, 15%, and 20% were 0.08, 0.12, 0.11, and 0.09, respectively; and the ground GI of 0%, 10%, 15%, and 20% were 0.03, 0.09, 0.09, and 0.10, respectively, indicating the installation of an interior landscape did not significantly impact high β waves.

Such findings are due to the intensity of illumination of artificial lighting being set at 500 lx, which is higher than the mean intensity of illumination of existing subway station space (355.89 lx) [17], to accord with the criteria of plant vegetation environment while building interior landscape models of subway stations. Therefore, the space with a brighter image than the actual subway station space was established, and stress was not expected to be induced, even with the interior landscape model with a GI of 0%.

### 3.2. Psychological Response to Different GI for the Interior Landscape of Subway Station Space

#### 3.2.1. Preference on Subway Interior Landscape Model with Different GI

One-way ANOVA was performed to investigate the preference of the subway interior landscape model with a different GI (Table 5). The underground GI of 15% and ground GI of 10% was highest at 4.48 points (SD = 0.70) and 4.50 points (SD = 0.62), respectively, followed by the underground GI of 10% with 4.05 points (SD = 0.67), the ground GI of 15% with 4.08 points (SD = 0.67), the underground GI of 20% with 3.67 points (SD = 0.73), and the ground GI of 20% with 3.57 points (SD = 0.79). 

The test result for homogeneity of variances showed that the *p*-value of the F value was 0.05 or below (*p*-value of underground subway station = 0.03, *p*-value of ground subway station = 0.031) and that the assumption of homogeneity of variance was not satisfied. For this reason, Games–Howell test was used to conduct a post-hoc test analysis (Table 6).

Meanwhile, the preferences of GI of 0% in the underground and ground were 2.40 points (SD = 0.89) and 2.92 points (SD = 0.94), respectively, indicating the preference for a subway station space with an interior landscape rather than for that with no interior landscape. The preference points of GI of 20% in the underground and ground were 3.67 points (SD = 0.73) and 3.57 points (SD = 0.79), respectively, suggesting that the implicit improvement of a GI is not preferred. 

These results are similar to those of previous studies [23,28], which claim that harmonious natural environmental elements including plant leaves become with other elements of whole space considering arrangement and shape has a more significant impact on the preference when introducing plants. Compared with the preferred level of the GI, users believe the interior landscape area with a GI of 15% is suitable; however, for ground subway stations, the model with a GI of 10% is preferred because of the effect and security of the inflow of natural light. Therefore, as ground subway stations are advantageous for securing natural light due to high floor heights, future studies on the required interior GI are needed. 

A cross-analysis was performed to investigate the recognition level of the interior landscape area of the subway interior landscape model with a different GI (Table 7). As a result, the underground GI of 15% and the ground GI of 10% received the highest response of “the interior landscape area is adequate” with 70.0% and 66.7%, respectively. Meanwhile, 53.3% and 55.0% of the respondents indicated that “the interior landscape area is adequate” for the underground GI of 10% and the ground GI of 15%, respectively. Of the respondents, 35.5% answered that “the interior landscape area is small” for the underground GI of 10%, while 43.3% of the respondents responded that “the interior landscape area is large” for the ground GI of 15%; this indicates that the recognition level of the interior landscape area differs depending on the availability of natural sunlight.

On the other hand, 76.6% and 86.7% of the respondents considered the interior landscape area as “large” and “very large”, respectively, in the case of GI of 20% in the underground and the ground, suggesting that 20% of GI in subway station spaces is considered excessive in terms of the plant density.

#### 3.2.2. Attention Restoration Effect of Subway Interior Landscape Models with Different GI

The one-way ANOVA scores of attention restoration effects for an underground and ground GI of 0% were 2.39 points (SD = 0.57) and 2.65 points (SD = 0.70), respectively (Table 8). Such results indicate that the attention restoration effect of subway station space with interior landscape is higher than that with no interior landscape. The result of the test for homogeneity of variances showed that the *p*-value of the F value was 0.05 or below (*p*-value of underground subway station = 0.09, *p*-value of ground subway station = 0.38) and that the assumption of homogeneity of variance was not satisfied. For this reason, the Games–Howell test was used to conduct a post-hoc test analysis (Table 9).

The underground GI of 15% showed the highest attention restoration effect of 4.42 points (SD = 0.72), whereas ground GI of 10%, 15%, and 20% were 4.22 (SD = 0.68), 4.39 (SD = 0.78), and 4.24 points (SD = 0.71), respectively. These results suggest that for the ground GI, only a 10% GI could lead to an attention restoration effect of 15–20% GI.

#### 3.2.3. Space Image of Subway Interior Landscape Model with Different GI

**Figure 4:**
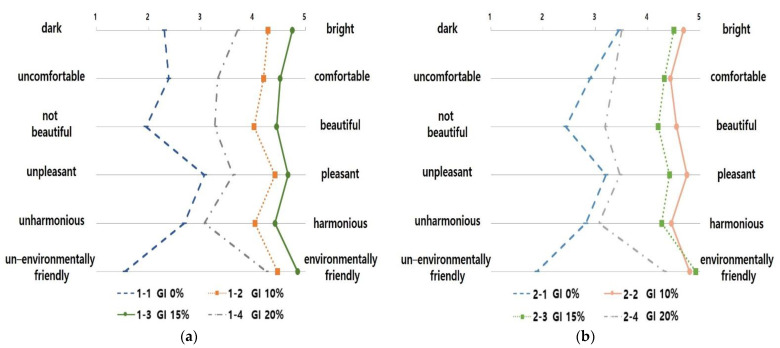
The Graph of Space Image Differences in Interior Landscape Models According to the GI: (**a**) The Graph of Space Image Differences in Underground Subway Interior Landscape Models According to the GI; (**b**) The Graph of Space Image Differences in Ground Subway Interior Landscape Models According to the GI.

The one-way ANOVA was conducted to evaluate space images of 3D landscape models of the subway interior according to the GI (Figure 4, Table 10 and Table 11). The test results for homogeneity of variances according to space image item showed that the *p*-value of the F value was 0.05 or below and that the assumption of homogeneity of variance was not satisfied. For this reason, the Games–Howell test was used to conduct a post-hoc test analysis (Table 12 and Table 13). As for the “dark–bright” image in underground subway stations, the GI of 15% was 4.75 points, being the highest. 

However, a GI of 10% and 15% were the most comfortable space images, with 4.20 (SD = 0.99) and 4.52 points (SD = 0.93), respectively, for “uncomfortable–comfortable.” The GI of 15% was 4.45 points (SD = 0.87) for “not beautiful–beautiful”, suggesting that it was the most beautiful space image. The GI of 10% and 15% were 4.42 points (SD = 0.87) and 4.67 points (SD = 0.66), respectively, for “unpleasant–pleasant”, indicating that they were the most pleasant space images. The GI of 10% and 15% resulted in 4.03 (SD = 1.29) and 4.42 points (SD = 0.98), respectively, for “unharmonious–harmonious”, indicating they were the most harmonious space images. The GI of 15% was found to be the most environmentally-friendly space image for “un-environmentally friendly–environmentally friendly.”

Regarding ground subway stations, the GI of 10% and 15% were the brightest space images with 4.68 (SD = 0.65) and 4.50 points (SD = 0.87), respectively, for “dark–bright.” The GI of 10% and 15% were the most positive images for “uncomfortable–comfortable” (4.43 points (SD = 0.87) and 4.55 points (SD = 0.67), respectively), “not beautiful–beautiful” (4.32 points (SD = 1.07) and 4.20 points (SD = 0.18), respectively) and “unpleasant–pleasant” (4.75 points (SD = 0.44) and 4.42 points (SD = 0.98), respectively). The GI of 10% and 15% were the most harmonious and environmentally friendly space images in terms of “unharmonious–harmonious” (4.45 points (SD = 0.93) and 4.80 points (SD = 0.51), respectively) and “un-environmentally friendly–environmentally friendly” (4.27 points (SD = 1.15) and 4.32 points (SD = 0.91), respectively).

In summary, for the underground subway station with no natural light introduced, the space image with a GI of 15% was most positively evaluated. Further, the ground subway station where natural light could be introduced, the model with a GI of 10% was most positively evaluated. These results agree with the results of a previous study [28], which concluded that the preference of GI is affected by comfort properties. 

Meanwhile, as the space with a GI of 15% was recognized as a more “environmentally friendly” space compared to that with a GI of 20% if it exceeds the optimum level, the effect of an “environmentally friendly” space image could be reduced. Therefore, considering the characteristics of a subway station interior space, unlike the exterior space, the interior landscape space should be established by considering the GI evaluated as pleasant and environmentally friendly by users.

#### 3.2.4. Correspondence Analysis on Subway Interior Landscape Models with Different GI

As shown in the correspondence analysis table (Table 14), this analysis was found to be statistically significant (*p* < 0.001). The one-dimensional R^2^ value was 41.6%, 2D R^2^ value was 35.4%, and 2D cumulative R^2^ value was 81.5%. Because a high relationship R^2^ value is considered when the 2D R^2^ is over 70% in correspondence analysis [57], the relationship between dimensions was significant. 

**Figure 5:**
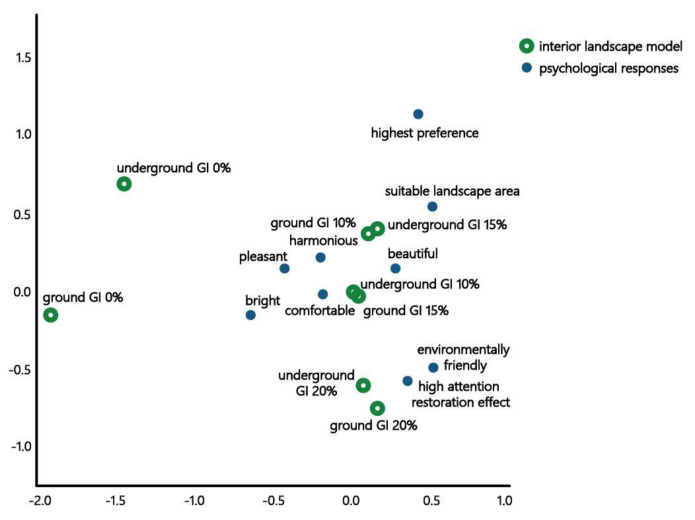
Positioning Map Between the Interior Landscape Model and Psychological Responses of the Subjects of the Survey According to the GI.

As presented in the positional map (Figure 5), which shows obtained results, “GI of 0% in the underground and ground”, “underground GI of 10%” and “ground GI of 15%”, “underground GI of 15%” and “ground GI of 10%”, and “GI of 20% in the underground and ground” were classified as the same group. In terms of “underground GI of 0%” and “ground GI of 0%”, the difference between models was larger than that of other groups. Furthermore, because “underground GI of 15%” and “ground GI of 10%” were classified as a similar group, space is recognized differently depending on the inflow of natural light. “GI of 20% in the underground and ground” were reclassified as “environmentally friendly” spaces providing “high attention restoration effect”: however, they had relatively low scores for “preference”, “suitable landscape area”, “pleasantness”, and “harmonious.” The “models of the underground and ground GI of 10–15%” were reclassified as “harmonious”, “beautiful”, “comfortable”, and “pleasant” spaces. Further, the “underground GI of 15%” and “ground GI of 10%” models had suitable landscape area and preferred it.

## 4. Discussion and Conclusions

This study provided reference data to prepare an interior landscape strategy for developing a subway station space by constructing a 3D interior landscape model, which considers the characteristics of subway station spaces and analyzes the psychological and physiological response of users about GI. 

Subway stations were classified as ground subway stations where natural light can be introduced and underground subway stations where natural light cannot be introduced to design interior landscapes considering the physical environments of subway stations.

Based on the research results, the optimum GI range for planning a pleasant and healthy interior landscape in a subway station space is summarized as follows.

First, based on the physiological response to the different GI of subway station spaces, the prefrontal α wave asymmetry value increased when the GI was 0% and 10–20%, regardless of the inflow of natural light (underground/ground), thereby suggesting that the installation of interior lighting evokes positive emotions.

Therefore, introducing an interior landscape in subway station space is necessary because the introduction of plants positively affects mental relaxation and psychological stability. Moreover, because psychophysiological stability and satisfaction is ensured by improving the GI in subway station spaces, it is expected to result in positive impacts such as environmental improvement and activation of subway station spaces.

Second, based on the psychological response with a different GI of the subway station space, the underground GI of 15% and the ground GI of 10% had the highest preference in the interior landscape model and the most suitable level of interior landscape installation area recognition. Furthermore, they were the most positively evaluated in all the space image adjective items. Such findings indicate that the GI of 10–15% is appropriate in interior landscape design for subway station spaces. In addition, based on detailed analysis using multidimensional scaling, the models of the underground GI of 15% and the ground GI of 10% were similar. They were the most preferred interior landscape models with the most suitable interior landscape area. This analysis shows that when plants are introduced in an indoor space, the degree of harmony between natural environmental elements (including plant leaves) with other elements in the space regarding arrangement, shape, and space characteristics has a more significant effect on the preference. Therefore, interior landscape planning must accommodate the space characteristics when plants are introduced in indoor spaces. 

Therefore, for underground subway stations with no natural lighting, a GI of 15% was the most suitable. Meanwhile, for the ground subway stations with natural lighting, the interior landscape with a GI of 10% was sufficient. 

Across the subway stations in Busan, the value of GI where an interior landscape is installed varies from a minimum of 1.03% to a maximum of 28.94% [17]. Furthermore, efforts to promote the implementation of and improve the interior landscape have been recently undertaken. Therefore, there is a pressing need for introducing interior landscapes with GIs falling within an appropriate range in consideration of the efficient use of subway station spaces, psychological satisfaction of the users, and cost of introduction and maintenance of the interior landscape.

According to a comparative analysis of the results of the present study and the previous related studies, a study that examined the physiological responses of users showed no difference in heart rate variability and EEG based on GI [21], which is similar to the EEG findings obtained in this study. It was indicated that the interior space with landscape induced users to feel more comfortable than the spaces without an interior landscape. Pertaining to psychological responses, in the study of [21], which examined the GI preference by setting up a laboratory having an area of 1.5 m × 1.5 m, the preference for a GI of 50% was found to be high. In another study that investigated GI preference in workspace [15], the GI of 10–20% was the most preferred, and when the GI is 20%, the attention restoration effect is high. Meanwhile, in this study, GI preference and attention restoration effect were the highest when the GI of 15% for underground subway stations and GI of 10% for ground subway stations. These findings indicate that different results are obtained depending on the target space of research.

Taken together, it is necessary to implement landscaping in indoor spaces for a positive emotional effect on users, with an emphasis on the interior landscape design considering the functional aspects of the space and the characteristics of the physical environment.

In this study, a differential GI response analysis was conducted using a 3D model according to the movement of users, such that the user experienced all the aspects of the interior landscape introduced in the subway station space. Our findings could serve to improve the existing GI measurement methods using 2D images and laboratory-based experiments. Furthermore, it can be argued that the proposed method is more effective in interior landscape design, taking into account the physical environment characteristics of subway stations.

This study has significance based on the following reasons. First, it implemented 3D landscape models of the subway interior by classifying subway stations as underground and ground subway stations according to the presence of natural light inflow in consideration of the physical properties of subway station spaces. Second, it evaluated responses of subway station users by implementing 3D landscape models of the subway interior instead of performing flat (2D) measurements as conducted in existing studies. Third, it divided subjects according to the GI as physiological responses and psychological responses to perform a more precise analysis and establish a direction for enhancing indoor environments of subway station spaces. Because the GI preference varies according to the presence of natural light inflow, it is important to develop an interior landscape plan by considering the characteristics of subway station spaces. In this regard, it is expected that the result of this study can provide data for developing an interior landscape plan for subway station spaces and enhancing such interior landscapes.

The limitations of this research are as follows. 

First, because the GI can be planned by utilizing various interior landscape techniques other than garden type/planter and wall type/pillar types, future studies on various interior landscape techniques are needed when introducing interior landscapes.

Second, research considering demographic characteristics should be conducted by sampling and investigating research subjects of all ages. 

Third, evaluation of the GI needs to be continuously performed by selecting various public space targets other than subway station spaces as research and expanding the research range. Therefore, further research on applying various interior landscape formation methods and accumulating research data with more research participants is required. It is anticipated that these processes will contribute to developing a more specific and feasible plan for implementing interior landscape in subway station spaces.

## Figures and Tables

**Table 1:** Image of Subway Interior Landscape Model according to GI.

Below /Above	Place	GI 0%	GI 10%	GI 15%	GI 20%
underground subway station	entrance	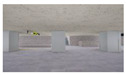	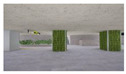	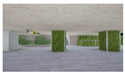	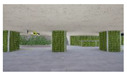
	square	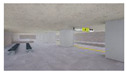	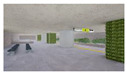	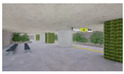	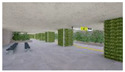
	passage	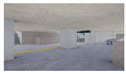	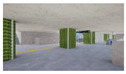	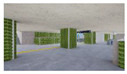	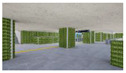
	faregate	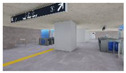	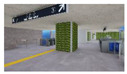	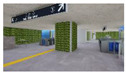	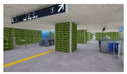
ground subway station	entrance	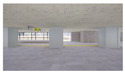	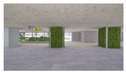	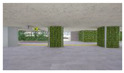	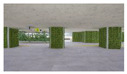
	square	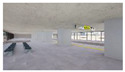	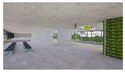	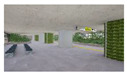	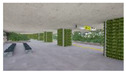
	passage	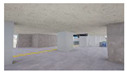	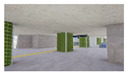	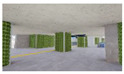	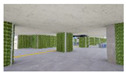
	faregate	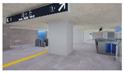	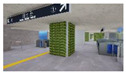	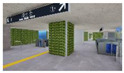	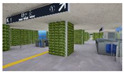

**Table 2:** Demographic Characteristics N(%).

Demographic Characteristics		Total
Gender	Male	30(50.0)
	Female	30(50.0)
	Total	60(100.0)
Age	20 s	30(50.0)
	30 s	30(50.0)
	Total	60(100.0)

**Table 3:** Prefrontal Alpha Asymmetry of Subway Interior Landscape Models with GI.

Interior Landscape Model		N	M(SD)	F	Post-hoc Test
underground subway station	eye-closed	60	0.45(0.92)	4.454 **	a
	GI 0%	60	0.47(0.90)		a
	GI 10%	60	0.96(0.99)		b
	GI 15%	60	0.97(0.81)		b
	GI 20%	60	0.87(1.05)		b
	total	300	0.75(0.99)		
ground subway station	eye-closed	60	0.45(0.92)	2.743 *	a
	GI 0%	60	0.64(0.80)		ab
	GI 10%	60	0.95((1.20)		b
	GI 15%	60	0.95(1.06)		b
	GI 20%	60	0.93(1.18)		b
	total	300	0.78(1.08)		

* *p* < 0.05, ** *p* < 0.01, F = between groups variance/within groups variance. Post-hoc test: Duncan test = a < ab < b.

**Table 4:** Prefrontal High β wave Asymmetry of Subway Interior Landscape Models with GI.

Interior Landscape Model		N	M(SD)	F
underground subway station	eye-closed	60	0.07(0.24)	0.387 (0.818)
	GI 0%	60	0.08(0.28)	
	GI 10%	60	0.12(0.29)	
	GI 15%	60	0.11(0.28)	
	GI 20%	60	0.09(0.28)	
	total	300	0.10(0.27)	
ground subway station	eye-closed	60	0.07(0.24)	0.363 (0.835)
	GI 0%	60	0.03(0.43)	
	GI 10%	60	0.09(0.33)	
	GI 15%	60	0.09(0.35)	
	GI 20%	60	0.10(0.31)	
	total	300	0.08(0.33)	

F = between groups variance/within groups variance.

**Table 5:** Analysis of Preferences of Interior Landscape Models in Subway according to GI.

Interior Landscape Model		N	M(SD)	F	Post-hoc Test
underground subway station	GI 0%	60	2.40(0.89)	85.420 ***	a
	GI 10%	60	4.05(0.67)		c
	GI 15%	60	4.48(0.70)		d
	GI 20%	60	3.67(0.73)		b
	total	240	3.65(1.08)		
ground subway station	GI 0%	60	2.92(0.94)	47.593 ***	a
	GI 10%	60	4.50(0.62)		d
	GI 15%	60	4.08(0.67)		c
	GI 20%	60	3.57(0.79)		b
	total	240	3.77(0.97)		

*** *p* < 0.001, F = between groups variance/within groups variance. Post-hoc test: Games–Howell test = a < b < c < d. Note. 5-point Likert scale, 1 = Strongly disagree, 2 = Disagree, 3 = Nomal, 4 = Agree, 5 = Strongly agree.

**Table 6:** Post-hoc Test for Interior Landscape Model Preference.

	(I) Model		GI 0%			GI 10%			GI 15%			GI 20%		
	(J) Model		GI 10%	GI 15%	GI 20%	GI 0%	GI 15%	GI 20%	GI 0%	GI 10%	GI 20%	GI 0%	GI 10%	GI 15%
underground subway station	Mean Difference (I–J)		−1.650 ***	−2.083 ***	−1.267 ***	1.650 ***	−0.433 **	0.383 *	2.083 ***	0.433 **	0.817 ***	1.267 ***	−0.383 *	−0.817 ***
	Std. Error		0.14	0.15	0.15	0.14	0.13	0.13	0.15	0.13	0.13	0.15	0.13	0.13
	95% Confidence Interval	Lower Bound	−2.03	−2.46	−1.65	1.27	−0.76	0.05	1.70	0.11	0.48	0.88	−0.72	−1.16
		Upper Bound	−1.27	−1.70	−0.88	2.03	−0.11	0.72	2.46	0.76	1.16	1.65	−0.05	−0.48
ground subway station	Mean Difference (I–J)		−1.583 ***	−1.167 ***	−0.650 ***	1.583 ***	0.417 **	0.933 ***	1.167 ***	−0.417 **	0.517 **	0.650 ***	−0.933 ***	−0.517 **
	Std. Error		0.15	0.15	0.16	0.15	0.12	0.13	0.15	0.12	0.13	0.16	0.13	0.13
	95% Confidence Interval	Lower Bound	−1.96	−1.56	−1.06	1.20	0.11	0.59	0.78	−0.73	0.17	0.24	−1.27	−0.87
		Upper Bound	−1.20	−0.78	−0.24	1.96	0.73	1.27	1.56	−0.11	0.87	1.06	−0.59	−0.17

* *p* < 0.05, ** *p* < 0.01, *** *p* < 0.001.

**Table 7:** Analysis of Level of Interior Landscape Area Recognition of Interior Landscape Models in Subway according to GI.

Interior Landscape Model		Too Low	Low	Suitable	High	Very High	Total	χ^2^
underground subway station	GI 10%	1	21	32	6	-	60	100.418 ***
		(1.7)	(35.0)	(53.3)	(10.0)		(100.0)	
	GI 15%	-	2	42	16	-	60	
			(3.3)	(70.0)	(26.7)		(100.0)	
	GI 20%	-	-	14	29	17	60	
				(23.3)	(48.3)	(28.3)	(100.0)	
	total	1	23	88	51	17	180	
		(0.6)	(12.8)	(48.9)	(28.3)	(9.4)	(100.0)	
ground subway station	GI 10%	-	-	40	2	-	60	120.948 ***
				(66.7)	(3.3)		(100.0)	
	GI 15%	-	-	33	26	1	60	
				(55.0)	(43.3)	(1.7)	(100.0)	
	GI 20%	-	-	8	30	22	60	
				(13.3)	(50.0)	(36.7)	(100.0)	
	total	-	18	81	58	23	180	
			(10.0)	(45.0)	(32.2)	(12.8)	(100.0)	

*** *p* < 0.001.

**Table 8:** Attention Restoration Effect of Interior Landscape Models in Subway According to GI.

Interior Landscape Model		N	M(SD)	F	Post-hoc Test
underground subway station	GI 0%	60	2.39(0.57)	100.832 ***	a
	GI 10%	60	4.04(0.72)		b
	GI 15%	60	4.42(0.72)		c
	GI 20%	60	4.24(0.71)		b
	total	240	3.78(1.09)		
ground subway station	GI 0%	60	2.65(0.70)	65.146 ***	a
	GI 10%	60	4.22(0.68)		b
	GI 15%	60	4.39(0.78)		b
	GI 20%	60	4.24(0.95)		b
	total	240	3.88(1.06)		

*** *p* < 0.001, F = between groups variance/within groups variance, Post-hoc test: Games-Howell test = a < b, Note. five-point Likert scale, 1 = Strongly disagree, 2 = Disagree, 3 = Nomal, 4 = Agree, 5 = Strongly agree.

**Table 9:** Post-hoc Test for Interior Landscape Model Attention Restoration Effect.

	(I) Model		GI 0%			GI 10%			GI 15%			GI 20%		
	(J) Model		GI 10%	GI 15%	GI 20%	GI 0%	GI 15%	GI 20%	GI 0%	GI 10%	GI 20%	GI 0%	GI 10%	GI 15%
underground subway station	Mean Difference (I–J)		−1.655 ***	−2.036 ***	−1.860 ***	1.655 ***	−0.381 *	−0.205	2.036 ***	0.381 *	0.176	1.860 ***	0.205	−0.176
	Std. Error		0.12	0.12	0.13	0.12	0.13	0.15	0.12	0.13	0.14	0.13	0.15	0.14
	95% Confidence Interval	Lower Bound	−1.96	−2.34	−2.21	1.35	−0.72	−0.58	1.73	0.04	−0.20	1.51	−0.17	−0.55
		Upper Bound	−1.35	−1.73	−1.51	1.96	−0.04	0.17	2.34	0.72	0.55	2.21	0.58	0.20
ground subway station	Mean Difference (I–J)		−1.567 ***	−1.740 ***	−1.590 ***	1.567 ***	−0.174	−0.024	1.740 ***	0.174	0.150	1.590 ****	0.024	−0.150
	Std. Error		0.13	0.14	0.15	0.13	0.13	0.15	0.14	0.13	0.16	0.15	0.15	0.16
	95% Confidence Interval	Lower Bound	−1.89	−2.09	−1.99	1.24	−0.52	−0.42	1.39	−0.18	−0.27	1.19	−0.37	−0.57
		Upper Bound	−1.24	−1.39	−1.19	1.89	0.18	0.37	2.09	0.52	0.57	1.99	0.42	0.27

* *p* < 0.05, *** *p* < 0.001, **** *p* < 0.0001.

**Table 10:** Space Image Difference Analysis of Interior Landscape Models in Underground Subway According to GI.

Space Image	Interior Landscape Model	N	M(SD)	F	Post-hoc Test
dark–bright	GI 0%	60	2.30(0.85)	85.713 ***	a
	GI 10%	60	4.28(0.96)		c
	GI 15%	60	4.75(0.54)		d
	GI 20%	60	3.70(1.11)		b
	total	240	3.76(1.28)		
uncomfortable –comfortable	GI 0%	60	2.38(0.83)	60.398 ***	a
	GI 10%	60	4.20(0.99)		c
	GI 15%	60	4.52(0.93)		c
	GI 20%	60	3.32(1.07)		b
	total	240	3.60(1.26)		
not beautiful –beautiful	GI 0%	60	1.95(0.83)	69.546 ***	a
	GI 10%	60	4.02(1.16)		c
	GI 15%	60	4.45(0.87)		d
	GI 20%	60	3.27(1.16)		b
	total	240	3.42(1.39)		
unpleasant –pleasant	GI 0%	60	3.07(0.73)	46.744 ***	a
	GI 10%	60	4.42(0.87)		c
	GI 15%	60	4.67(0.66)		c
	GI 20%	60	3.62(1.03)		b
	total	240	3.94(1.05)		
unharmonious –harmonious	GI 0%	60	2.68(0.91)	33.054 ***	a
	GI 10%	60	4.03(1.29)		b
	GI 15%	60	4.42(0.98)		b
	GI 20%	60	3.07(1.15)		a
	total	240	3.55(1.30)		
un-environmentally friendly –environmentally friendly	GI 0%	60	1.55(0.79)	231.781 ***	a
	GI 10%	60	4.47(0.93)		b
	GI 15%	60	4.85(0.48)		c
	GI 20%	60	4.25(0.79)		b
	total	240	3.78(1.51)		

*** *p* < 0.001, F = between groups variance/within groups variance, post-hoc test: Games–Howell test = a < b < c < d.

**Table 11:** Space Image Difference Analysis of Interior Landscape Models in Ground Subway according to GI.

Space Image	Interior Landscape Model	N	M(SD)	F	Post-hoc Test
dark–bright	GI 0%	60	3.45(1.11)	25.105 ***	a
	GI 10%	60	4.68(0.65)		b
	GI 15%	60	4.50(0.87)		b
	GI 20%	60	3.50(1.27)		a
	total	240	4.03(1.15)		
uncomfortable –comfortable	GI 0%	60	2.90(0.88)	35.081 ***	a
	GI 10%	60	4.43(0.87)		b
	GI 15%	60	4.32(1.07)		b
	GI 20%	60	3.35(1.07)		a
	total	240	3.75(1.17)		
not beautiful –beautiful	GI 0%	60	2.43(0.87)	58.371 ***	a
	GI 10%	60	4.55(0.67)		c
	GI 15%	60	4.20(1.18)		c
	GI 20%	60	3.18(1.11)		b
	total	240	3.59(1.28)		
unpleasant –pleasant	GI 0%	60	3.20(0.92)	41.435 ***	a
	GI 10%	60	4.75(0.44)		b
	GI 15%	60	4.42(0.98)		b
	GI 20%	60	3.47(1.10)		a
	total	240	3.96(1.10)		
unharmonious –harmonious	GI 0%	60	2.83(0.87)	36.839 ***	a
	GI 10%	60	4.45(0.93)		b
	GI 15%	60	4.27(1.15)		b
	GI 20%	60	3.07(1.23)		a
	total	240	3.65(1.27)		
un-environmentally friendly –environmentally friendly	GI 0%	60	1.88(0.74)	282.180 ***	a
	GI 10%	60	4.80(0.51)		c
	GI 15%	60	4.92(0.28)		c
	GI 20%	60	4.32(0.91)		b
	total	240	3.98(1.40)		

*** *p* < 0.001, F = between groups variance/within groups variance, post-hoc test: Games–Howell test = a < b < c < d.

**Table 12:** Post-hoc Test Analysis for Interior Landscape Model Space Image in Underground Subway.

	(I) Model		GI 0%			GI 10%			GI 15%			GI 20%		
	(J) Model		GI 10%	GI 15%	GI 20%	GI 0%	GI 15%	GI 20%	GI 0%	GI 10%	GI 20%	GI 0%	GI 10%	GI 15%
dark–bright	Mean Difference (I–J)		−1.983 ***	−2.450 ***	−1.400 ***	1.983 ***	−0.467 *	0.583 *	2.450 ***	0.467 *	1.050 ***	1.400 *	−0.583 *	−1.050 ***
	Std. Error		0.17	0.13	0.18	0.17	0.14	0.19	0.13	0.14	0.16	0.18	0.19	0.16
	95% Confidence Interval	Lower Bound	−2.41	−2.79	−1.87	1.55	−0.84	0.09	2.11	0.10	0.63	0.93	−1.08	−1.47
		Upper Bound	−1.55	−2.11	−0.93	2.41	−0.10	1.08	2.79	0.84	1.47	1.87	−0.09	−0.63
uncomfortable –comfortable	Mean Difference (I–J)		−1.817 ***	−2.133 ***	−0.933 ***	1.817 ***	−0.317	0.883 *	2.133 *	0.317	1.200 *	0.933 *	−0.883 *	−1.200 *
	Std. Error		0.17	0.16	0.17	0.17	0.18	0.19	0.16	0.18	0.18	0.17	0.19	0.18
	95% Confidence Interval	Lower Bound	−2.25	−2.55	−1.39	1.38	−0.77	0.39	1.72	−0.14	0.72	0.48	−1.37	−1.68
		Upper Bound	−1.38	−1.72	−0.48	2.25	0.14	1.37	2.55	0.77	1.68	1.39	−0.39	−0.72
not beautiful –beautiful	Mean Difference (I–J)		−2.067 ***	−2.500 ***	−1.317 ***	2.067 ***	−0.433	0.750 **	2.500 ***	0.433	1.183 ***	1.317 ***	−0.750 **	−1.183 ***
	Std. Error		0.18	0.16	0.18	0.18	0.19	0.21	0.16	0.19	0.19	0.18	0.21	0.19
	95% Confidence Interval	Lower Bound	−2.55	−2.91	−1.80	1.59	−0.92	0.20	2.09	−0.05	0.69	0.83	−1.30	−1.67
		Upper Bound	−1.59	−2.09	−0.83	2.55	0.05	1.30	2.91	0.92	1.67	1.80	−0.20	−0.69
unpleasant –pleasant	Mean Difference (I–J)		−1.350 ***	−1.600 ***	−0.550 **	1.350 ***	−0.250	0.800 ***	1.600 ***	0.250	1.050 ***	0.550 **	−0.800 ***	−1.050 ***
	Std. Error		0.15	0.13	0.16	0.15	0.14	0.17	0.13	0.14	0.16	0.16	0.17	0.16
	95% Confidence Interval	Lower Bound	−1.73	−1.93	−0.98	0.97	−0.62	0.35	1.27	−0.12	0.64	0.12	−1.25	−1.46
		Upper Bound	−0.97	−1.27	−0.12	1.73	0.12	1.25	1.93	0.62	1.46	0.98	−0.35	−0.64
unharmonious –harmonious	Mean Difference (I–J)		−1.350 ***	−1.733 ***	−0.383	1.350 ***	−0.383	0.967 ***	1.733 ***	0.383	1.350 ***	0.383	−0.967 ***	−1.350 ***
	Std. Error		0.20	0.17	0.19	0.20	0.21	0.22	0.17	0.21	0.19	0.19	0.22	0.19
	95% Confidence Interval	Lower Bound	−1.88	−2.18	−0.88	0.82	−0.93	0.39	1.28	−0.16	0.84	−0.11	−1.55	−1.86
		Upper Bound	−0.82	−1.28	0.11	1.88	0.16	1.55	2.18	0.93	1.86	0.88	−0.39	−0.84
un-environmentally friendly –environmentally friendly	Mean Difference (I–J)		−2.917 ***	−3.300 ***	−2.700 ***	2.917 ***	−0.383 *	0.217	3.300 ***	0.383 *	0.600 ***	2.700 ***	−0.217	−0.600 ***
	Std. Error		0.16	0.12	0.14	0.16	0.14	0.16	0.12	0.14	0.12	0.14	0.16	0.12
	95% Confidence Interval	Lower Bound	−3.33	−3.61	−3.08	2.51	−0.74	−0.19	2.99	0.03	0.29	2.32	−0.63	−0.91
		Upper Bound	−2.51	−2.99	−2.32	3.33	−0.03	0.63	3.61	0.74	0.91	3.08	0.19	−0.29

* *p* < 0.05, ** *p* < 0.01, *** *p* < 0.001.

**Table 13:** Post-hoc Test Analysis for Interior Landscape Model Space Image in Ground Subway.

	(I) Model		GI 0%			GI 10%			GI 15%			GI 20%		
	(J) Model		GI 10%	GI 15%	GI 20%	GI 0%	GI 15%	GI 20%	GI 0%	GI 10%	GI 20%	GI 0%	GI 10%	GI 15%
dark–bright	Mean Difference (I–J)		−1.233 ***	−1.050 ***	−0.050	1.233 ***	0.183	1.183 ***	1.050 ***	−0.183	1.000 ***	0.050	−1.183 ***	−1.000 ***
	Std. Error		0.17	0.18	0.22	0.17	0.14	0.18	0.18	0.14	0.20	0.22	0.18	0.20
	95% Confidence Interval	Lower Bound	−1.67	−1.53	−0.62	0.80	−0.18	0.70	0.57	−0.55	0.48	−0.52	−1.67	−1.52
		Upper Bound	−0.80	−0.57	0.52	1.67	0.55	1.67	1.53	0.18	1.52	0.62	−0.70	−0.48
uncomfortable –comfortable	Mean Difference (I–J)		−1.533 ***	−1.417 ***	−0.450	1.533 ***	0.117	1.083 ***	1.417 ***	−0.117	0.967 ***	0.450	−1.083 ***	−0.967 ***
	Std. Error		0.16	0.18	0.18	0.16	0.18	0.18	0.18	0.18	0.20	0.18	0.18	0.20
	95% Confidence Interval	Lower Bound	−1.95	−1.88	−0.92	1.12	−0.35	0.62	0.95	−0.58	0.46	−0.02	−1.55	−1.47
		Upper Bound	−1.12	−0.95	0.02	1.95	0.58	1.55	1.88	0.35	1.47	0.92	−0.62	−0.46
not beautiful –beautiful	Mean Difference (I–J)		−2.117 ***	−1.767 ***	−0.750 ***	2.117 ***	0.350	1.367 ***	1.767 ***	−0.350	1.017 ***	0.750 ***	−1.367 ***	−1.017 ***
	Std. Error		0.14	0.19	0.18	0.14	0.18	0.17	0.19	0.18	0.21	0.18	0.17	0.21
	95% Confidence Interval	Lower Bound	−2.49	−2.26	−1.23	1.75	−0.11	0.93	1.27	−0.81	0.47	0.27	−1.81	−1.56
		Upper Bound	−1.75	−1.27	−0.27	2.49	0.81	1.81	2.26	0.11	1.56	1.23	−0.93	−0.47
unpleasant –pleasant	Mean Difference (I–J)		−1.550 ***	−1.217 ***	−0.267	1.550 ***	0.333	1.283 ***	1.217 ***	−0.333	0.950 ***	0.267	−1.283 ***	−0.950 ***
	Std. Error		0.13	0.17	0.18	0.13	0.14	0.15	0.17	0.14	0.19	0.18	0.15	0.19
	95% Confidence Interval	Lower Bound	−1.89	−1.67	−0.75	1.21	−0.03	0.88	0.77	−0.70	0.46	−0.21	−1.68	−1.44
		Upper Bound	−1.21	−0.77	0.21	1.89	0.70	1.68	1.67	0.03	1.44	0.75	−0.88	−0.46
unharmonious–harmonious	Mean Difference (I–J)		−1.633 ***	−1.450 ***	−0.250	1.633 ***	0.183	1.383 ***	1.450 ***	−0.183	1.200 ***	0.250	−1.383 ***	−1.200 ***
	Std. Error		0.16	0.19	0.20	0.16	0.19	0.20	0.19	0.19	0.22	0.20	0.20	0.22
	95% Confidence Interval	Lower Bound	−2.06	−1.94	−0.76	1.20	−0.31	0.86	0.96	−0.68	0.63	−0.26	−1.90	−1.77
		Upper Bound	−1.20	−0.96	0.26	2.06	0.68	1.90	1.94	0.31	1.77	0.76	−0.86	−0.63
un-environmentally friendly –environmentally friendly	Mean Difference (I–J)		−2.917 ***	−3.033 ***	−2.433 ***	2.917 ***	−0.117	0.483 **	3.033 ***	0.117	0.600 ***	2.433 ***	−0.483 **	−0.600 ***
	Std. Error		0.12	0.10	0.15	0.12	0.08	0.14	0.10	0.08	0.12	0.15	0.14	0.12
	95% Confidence Interval	Lower Bound	−3.22	−3.30	−2.83	2.61	−0.31	0.13	2.77	−0.08	0.28	2.04	−0.84	−0.92
		Upper Bound	−2.61	−2.77	−2.04	3.22	0.08	0.84	3.30	0.31	0.92	2.83	−0.13	−0.28

** *p* < 0.01, *** *p* < 0.001.

**Table 14:** A Correspondence Analysis Table between the Interior Landscape Model and Psychological Responses of the Subjects of the Survey According to the GI.

Dimension	Singular Value	Inertia	Chi Square	Sig.	Proportion of Inertia		Confidence Singular Value	
					Accounted for	Cumulative	Standard Deviation	Correlation 2
1	0.188	0.035	-	-	0.461	0.461	0.017	0.047
2	0.165	0.027	-	-	0.354	0.815	0.019	-
3	0.106	0.011	-	-	0.146	0.961	-	-
4	0.044	0.002	-	-	0.026	0.987	-	-
5	0.029	0.001	-	-	0.011	0.997	-	-
6	0.014	0.000	-	-	0.002	1.000	-	-
7	0.003	0.000	-	-	0.000	1.000	-	-
Total	-	0.076	175.024	0.000 a	1.000	1.000	-	-

a. 56 degrees of freedom.

## Data Availability

Data sharing not applicable.
